# Stepwise Splitting of Ribosomal Proteins from Yeast Ribosomes by LiCl

**DOI:** 10.1371/journal.pone.0101561

**Published:** 2014-07-03

**Authors:** Kerli Piir, Tiina Tamm, Ivan Kisly, Triin Tammsalu, Jaanus Remme

**Affiliations:** Institute of Molecular and Cell Biology, University of Tartu, Tartu, Estonia; The Scripps Research Institute, United States of America

## Abstract

Structural studies have revealed that the core of the ribosome structure is conserved among ribosomes of all kingdoms. Kingdom-specific ribosomal proteins (r-proteins) are located in peripheral parts of the ribosome. In this work, the interactions between rRNA and r-proteins of eukaryote *Saccharomyces cerevisiae* ribosome were investigated applying LiCl induced splitting and quantitative mass spectrometry. R-proteins were divided into four groups according to their binding properties to the rRNA. Most yeast r-proteins are removed from rRNA by 0.5–1 M LiCl. Eukaryote-specific r-proteins are among the first to dissociate. The majority of the strong binders are known to be required for the early ribosome assembly events. As compared to the bacterial ribosome, yeast r-proteins are dissociated from rRNA at lower ionic strength. Our results demonstrate that the nature of protein-RNA interactions in the ribosome is not conserved between different kingdoms.

## Introduction

Recent advances in determining the structure of eukaryotic ribosome [1]–[3] open new possibilities to analyse structure-function relation of the individual ribosome components. Ribosomal proteins (r-proteins) are important players in both ribosome structure and function. Conservation of ribosome structure throughout the three kingdoms allows predicting the function of some eukaryotic r-proteins using their bacterial homologues. Out of 79 r-proteins in eukaryotes, 35 are evolutionarily conserved in all kingdoms of life, 32 are shared between eukaryotes and archaea, and 12 are eukaryote-specific [4], [5]. Over 50 years of studies mostly on the bacterial ribosome have revealed that binding of translation factors, tRNA, and mRNA to the ribosome is largely guided by r-proteins [6].

Splitting of ribosomal proteins by high salt has proven to be a useful approach to study bacterial ribosomes. It has been observed that each r-protein dissociates from the ribosomes at specific LiCl concentration [7]. Splitting of r-proteins at increasing LiCl concentration is in reverse order of association of r-proteins during ribosome assembly [7], [8]. It is interesting to compare functional importance of eukaryotic r-proteins with the prokaryotic homologs having structural information about both systems. It is also worth to test whether the interactions between rRNA and r-proteins are similar for homologous proteins. One way to test the nature of RNA-protein interactions is to analyse the resistance of the ribonucleoprotein complex to salt perturbation. Dissociation pattern of r-proteins of yeast ribosome large subunit has previously been analysed using semi-quantitative two-dimensional electrophoresis [9]. However, applying metabolically labeled r-proteins together with mass spectrometry allows the detection of yeast r-protein splitting under LiCl in a quantitative manner.

## Materials and Methods

### Yeast strains and growth conditions for SILAC labeling

*Saccharomyces cerevisiae* haploid strain TYSC110 (*MATa his3Δ1 leu2Δ0 ura3Δ0 met15Δ0 lys2Δ0 arg4Δ::kanMX4*) was constructed by crossing EUROSCARF strains BY4742 (*MATα his3Δ1 leu2Δ0 ura3Δ0 lys2Δ0*) and *arg4Δ* (*MATa his3Δ1 leu2Δ0 ura3Δ0 met15Δ YHR018c::kanMX4*) followed by diploid isolation, sporulation, tetrad dissection and selection of a lysine and arginine auxotroph.

Yeast cells were grown at 30°C in „light“ synthetic minimal (SD) media (0.67% yeast nitrogen base without amino acids, 2% glucose) supplemented with 20 mg/l L-histidine, 60 mg/l L-leucine, 20 mg/l L-methionine, 20 mg/l uracil, 30 mg/l L-lysine and 20 mg/l L-arginine [10] to mid log phase (OD_600_ of 1.8).

For lysine and arginine double SILAC labeling [11], L-lysine and L-arginine in the media were substituted with isotopically heavy 30 mg/l ^13^C_6_^15^N_2 _L-lysine and 20 mg/l ^13^C_6_^15^N_4 _L-arginine (Cambridge Isotope Laboratories), respectively. Cells were grown in “heavy” medium at 30°C for 16 h (approximately 10 generations) to mid log phase (OD_600_ of 1.4).

### Isolation of “light” and “heavy” ribosomes

Normal and SILAC-labeled yeast cells were collected by centrifugation at 4500 rpm for 10 min at 4°C, washed with cold deionized water and resuspended in 20 ml of lysis buffer (20 mM Tris-HCl pH 7.5, 140 mM NaCl, 5 mM MgCl_2_, 1 mM DTE) containing protease inhibitors (1 mM PMSF, 2 µg/ml aprotinin, 5 µg/ml pepstatin A, 1 µg/ml leupeptin). Cells were lysed with 5 to 6 cycles in French Press (Thermo Electron Corporation) at 18000 psi. Cell lysate was centrifuged twice at 15500 rpm for 15 min at 4°C in Type 50.2 Ti rotor (Beckmann). The supernatant was collected and the ribosomal particles were pelleted by centrifugation through a 20% sucrose cushion in buffer A (20 mM Tris-HCl pH 7.5, 250 mM NaCl, 5 mM MgCl_2_, 1 mM DTE) at 35000 rpm for 24 h (ω^2^t = 1.16*10^12^) at 4°C in in the same rotor. The pellet was resuspended in 2 ml of buffer A at 4°C for at least 2 hours, cleared at 15000 rpm for 20 min at 4°C in the same rotor and absorbance at 260 nm (A_260_) was measured. Thirty-five to forty A_260_ units (U) of ribosomal particles was carried onto a 10%–30% (w/w) sucrose gradient in buffer A and centrifuged at 20500 rpm for 16 h 15 min (ω^2^t = 2.7*10^11^) at 4°C in SW28 rotor (Beckmann). Fractions containing 80S ribosomal particles were collected, diluted 2 times with buffer A and concentrated using 100 000 Da MwCo filters (Millipore). Ribosomes were frozen in liquid nitrogen and stored at –80°C.

### Splitting of ribosomal proteins and sample preparation

Six A_260_ units of “light” 80S ribosomes in buffer A was mixed with 8 M LiCl in buffer A to a final concentration of 0 M, 0.5 M, 1 M or 2 M LiCl, respectively. Ribosomal particles were shaken at 12 rpm for 15 h at 4°C and pelleted by centrifugation at 80000 rpm for 2 h at 4°C in TLA-120.1 rotor (Beckmann).

Following the centrifugation, ⅓ of the supernatant fraction containing split proteins was mixed with one A_260_ unit of “heavy” 80S ribosomes in buffer A and proteins were precipitated for overnight at 4°C by adding TCA to a final concentration of 10%. Split protein pellet was washed with cold 80% acetone and resuspended in 7 M urea: 2 M thiourea solution. The core protein pellet yielding from centrifugation step was resuspended in 7 M urea: 2 M thiourea solution and ⅓ of it was mixed with one A_260_ unit of “heavy” 80S ribosomes in buffer A.

Thereafter, proteins in both split and core protein fractions were reduced for 1 h at RT by adding 1 mM DTT and carbamidomethylated with 5 mM iodoacetamide for 1.5 h at RT in the dark. Proteins were digested with endoproteinase Lys-C (Wako) at an enzyme to protein ratio 1∶50 for 4 h at RT. Following digestion, ½ of each sample was left at RT for overnight. The urea concentration in the other ½ of the solutions was reduced by adding 4 volumes of 100 mM NH_3_HCO_3_ and peptides were further digested with mass spectrometry grade trypsin (Promega; enzyme to protein ratio 1∶100) at RT for overnight. Enzymes were inactivated by addition of TFA to a final concentration of 1%.

### Subunit dissociation assay of 80S Ribosomes

Six A_260_ units of “light” 80S ribosomes in buffer A containing 0 M, 0.5 M, 1 M or 2 M LiCl were loaded onto linear 10–30% (w/w) sucrose gradients in buffer A and centrifuged at 22500 rpm for 16 h (ω^2^t = 3.2*10^11^) at 4°C in SW28 rotor (Beckmann). Gradients were collected from the bottom and ribosome profiles were monitored at 260 nm.

### LC-MS/MS analysis

Two independent biological replicates and two technical replicates were analysed. Peptides were desalted on self-made reverse-phase C_18_ stop and go extraction tips [12] and analysed by LC-MS/MS with LTQ-Orbitrap (Thermo Scientific) coupled to an Agilent 1200 nanoflow LC via nanoelectrospray ion source (Proxeon). One µg of purified peptides were injected at a flow rate of 700 nl/min into 75 µm×150 mm fused silica emitter (Proxeon), packed in-house with Reprosil-Pur 120 C18-AQ, 3 µm (Dr. Maisch GmbH) stationary phase, and eluted with 150 minute linear gradient of 3% to 40% of solvent B (80% acetonitrile, 0.5% acetic acid) in solvent A (0.5% acetic acid) at a flow rate of 200 nl/min. The LTQ-Orbitrap was operated in data dependent mode and a “lock mass” option was enabled for m/z 445.120030 to improve mass accuracy. Precursor ion full scan spectra (m/z 300 to 1800) were acquired in the orbitrap in profile with a resolution 60000 at m/z 400 (target value of 1 000 000 ions, maximum injection time 500 ms). Five most intense ions were fragmented in linear ion trap by collision-induced dissociation (normalised collision energy 35.0%) and spectra were acquired in centroid (target value of 5000 ions, maximum injection time 150 ms). Dynamic exclusion option was enabled (exclusion duration 120 s), and ions with unassigned charge state and singly charged ions were rejected.

### Data analysis

Raw mass spectrometric data files were processed using MaxQuant software (version 1.1.1.36) [13] and searched against the *Saccharomyces* Genome Database protein sequences including protein translations of all systematically named ORFs (SGD, http://www.yeastgenome.org; downloaded in February 2009). Enzyme specificity was set to Lys-C or trypsin for samples digested with Lys-C only or Lys-C and trypsin, respectively. Cleavage at N-terminal to proline residues was allowed. Carbamidomethylation of cysteines was selected as fixed modification, and oxidation of methionines and acetylation of protein N-termini were selected as variable modifications. A maximum number of two missed cleavages were allowed, a minimum peptide length was set to six amino acids, false discovery rate of 1% was set as a threshold at both protein and peptide level, and mass deviation of 6 ppm was set for main search and 0.5 Da for MS/MS peaks. Lys^8^ and Arg^10^ were selected as heavy labels and minimum of two SILAC pairs had to be counted for quantification. The percentage of each protein in the split or core protein fraction at different LiCl concentrations was calculated from the “heavy” to “light” ratios. The amount of a protein in both split and core fraction together at given LiCl concentration was taken as 100%. The number of peptides identified for quantitation, average percentage values in core fraction with standard deviations for each r-protein are presented in Table S1. Table S1 also includes new nomenclature for r-proteins as proposed by [14].

## Results and Discussion

**Figure 1:**
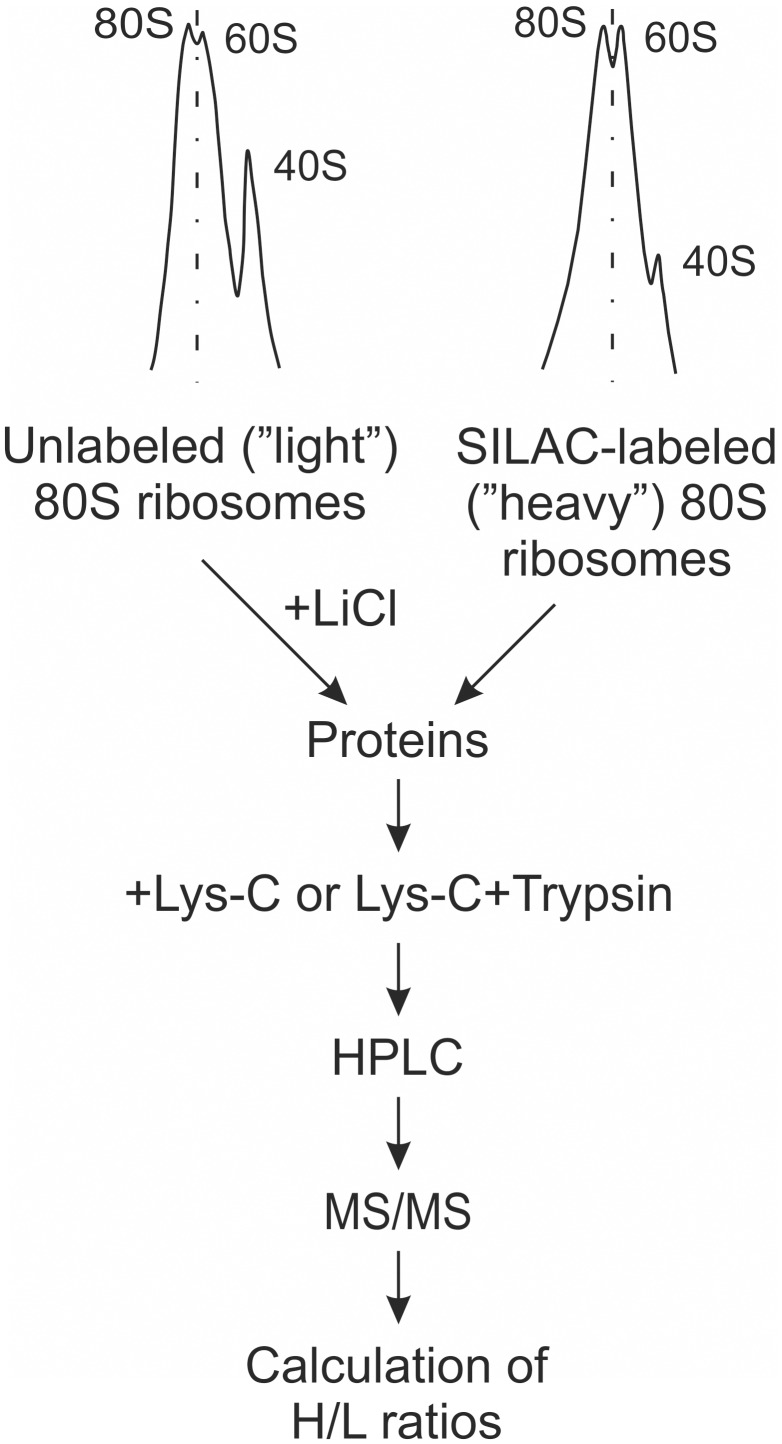
Schematic overview of the experimental approach. Yeast cells were grown in media containing either normal (“light”) or labeled (“heavy”) amino acids. Cells were lysed and “light” and “heavy” 80S ribosomes were collected by sucrose gradient centrifugation. After incubation of “light” 80S ribosomes with different concentrations of LiCl, core and supernatant fractions were separated by centrifugation and mixed with ”heavy” ribosomes. Ribosomal proteins were hydrolysed with Lys-C or with Lys-C + trypsin and peptides were separated by HPLC. “Heavy” to “light” ratio of peptides was determined by MS/MS for each protein.

In order to analyse the dissociation of yeast 80S ribosomes by different LiCl concentrations the stable isotope labeling by amino acids in cell culture (SILAC) in combination with mass spectrometry was applied (Figure 1). Haploid *lys2Δ arg4Δ* cells were grown in media containing either normal (“light”) or labeled (“heavy”) L-lysine and L-arginine. Two populations of ribosomes (“light” and “heavy”) were isolated by centrifugation in a sucrose density gradient.

“Light” ribosomes were incubated in the presence of different concentrations of LiCl. At this stage, r-proteins referred to as split proteins, dissociate from the ribosomes. The proteins associated with rRNA represent core proteins at particular LiCl concentration. The ribosomal core and supernatant fractions were separated by centrifugation. “Heavy” 80S ribosomes were added to each fraction followed by digestion of proteins by endoproteinases Lys-C alone or with combination with trypsin. “Heavy” to “light” ratio of peptides was determined by HPLC-MS/MS for each protein (except for rpL39, rpL40 and rpL41).

**Figure 2:**
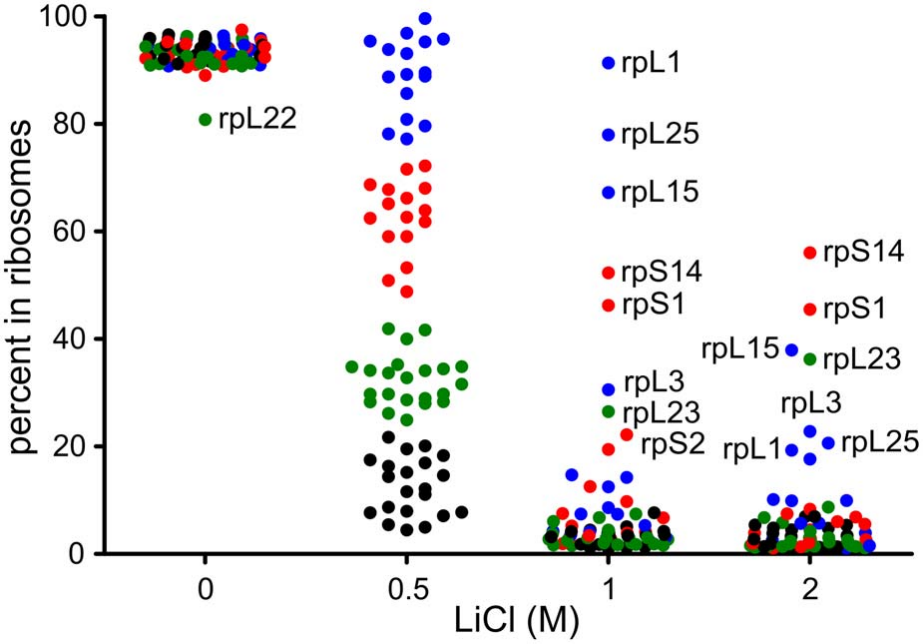
Splitting of ribosomal proteins from yeast ribosomes by LiCl. “Light” yeast 80S ribosomes were incubated with 0 M, 0.5 M, 1 M or 2 M LiCl, whereafter core and supernatant fractions were separated by centrifugation and “heavy” ribosomes were added to each fraction. Ribosomal proteins were precipitated with TCA, hydrolysed with Lys-C or with Lys-C + trypsin and peptides were separated by HPLC. “Heavy” to “light” ratio of peptides was determined by MS/MS for each protein. Proteins are grouped according to their amount in the core fraction at 0.5 M LiCl. Group A (blue) – 75–100%, B (red) – 48–75%, C (green) – 22–48% and D (black) – 0–22% in the core.

LiCl concentrations of 0 M, 0.5 M, 1 M and 2 M were used to split r-proteins. Effect of LiCl on the ribosome subunit association was tested. Addition of LiCl leads to the dissociation of the 80S ribosome into 40S and 60S subunits (Figure S1). Thus, LiCl induced splitting of r-proteins was analysed from ribosomal subunits rather than 80S ribosomes. At 0 M LiCl, all the r-proteins remained associated to the ribosome except for protein rpL22, of which 19.2% dissociated from the ribosome (Figure 2, Table S1). Large fraction of r-proteins was removed from the ribosome already at 0.5 M LiCl concentration. R-proteins were thus divided into four groups according to the extent of dissociation at this concentration: group A - more than 75%, B - 48–75%, C - 22–48%, and D - less than 22% in the core. Consequently, 21 r-proteins belong to the group D, 22 r-proteins constitute group C, groups A and B both contain 16 r-proteins (Table 1).

**Table 1:** Grouping of yeast r-proteins according to fraction of each protein in the ribosomal core at given LiCl concentration.

In core:	0–22%	22–48%	48–75%	75–100%
**0.5 M LiCl**	rpS0, rpS6, rpS7, rpS17,rpS21, rpS22, rpS24,rpS26, rpS27, rpS28, rpS30	rpS3, rpS4,rpS5, rpS9,rpS10, rpS12, rpS15, rpS16, rpS19, rpS20,rpS25, rpS29	rpS1, rpS2,rpS8, rpS14,rpS18, rpS23,rpS31	rpS11, rpS13
	rpL7, rpL9, rpL11, rpL26,rpL29, rpL31, rpL35, rpL38,rpL42, rpL43	rpL2, rpL4,rpL6, rpL14,rpL17, rpL20,rpL22, rpL23,rpL24, rpL32	rpL5, rpL12,rpL16, rpL21,rpL27, rpL30,rpL33, rpL34,rpP0	rpL1, rpL3, rpL8,rpL10, rpL13,rpL15, rpL18,rpL19, rpL25,rpL28, rpL36,rpL37, rpP1, rpP2
**1 M LiCl**	rpS0, rpS3, rpS4, rpS5, rpS6,rpS7, rpS8, rpS9, rpS10, rpS11,rpS12, rpS13, rpS15, rpS16,rpS17, rpS18, rpS19, rpS20,rpS21, rpS22, rpS23, rpS24,rpS25, rpS26, rpS27, rpS28,rpS29, rpS30, rpS31	rpS1, rpS2	rpS14	
	rpL2, rpL4, rpL5, rpL6, rpL7,rpL8, rpL9, rpL10, rpL11,rpL12, rpL13, rpL14, rpL16,rpL17, rpL18, rpL19, rpL20,rpL21, rpL22, rpL24, rpL26,rpL27, rpL28, rpL29, rpL30,rpL31, rpL32, rpL33, rpL34,rpL35, rpL36, rpL37, rpL38,,rpL42, rpL43, rpP0,rpP1, rpP2	rpL3, rpL23	rpL15	rpL1, prL25
**2 M LiCl**	rpS0, rpS2, rpS3, rpS4, rpS5,rpS6, rpS7, rpS8, rpS9, rpS10,rpS11, rpS12, rpS13, rpS15,rpS16, rpS17, rpS18, rpS19,rpS20, rpS21, rpS22, rpS23,rpS24, rpS25, rpS26, rpS27,rpS28, rpS29, rpS30, rpS31	rpS1	rpS14	
	rpL1, rpL2, rpL4, rpL5, rpL6,rpL7, rpL8, rpL9, rpL10, rpL11,rpL12, rpL13, rpL14, rpL16,rpL17, rpL18, rpL19, rpL20,rpL21, rpL22, rpL24, rpL25,rpL26, rpL27, rpL28, rpL29,rpL30, rpL31, rpL32, rpL33,rpL34, rpL35, rpL36, rpL37,rpL38,, rpL42, rpL43, rpP0,rpP1, rpP2	rpL3,rpL15, rpL23		

At 1 M LiCl concentration, most of the r-proteins were removed from the ribosome by more than 80% leaving only proteins rpS14, rpL1, rpL15 and rpL25 in the core fraction in significant amounts (50–90%) and rpS1, rpL3 and rpL23 in the core in lower levels (20–50%) (Figure 2, Table S1). When ribosomes were treated with 2 M LiCl, rpS1, rpS14, rpL3, rpL15 and rpL23 remained associated with the rRNA by about the same level, whereas the amount of rpL1 and rpL25 in the core reduced significantly. Moreover, some proteins, e. g. rpS1, rpS14 and rpL23, remained in the core fraction to slightly higher extent even at 4 M LiCl concentrations (data not shown). Thus, most of the yeast ribosomal proteins dissociate from the ribosome at a specific salt concentration.

Splitting of yeast *S. carlsbergensis* large ribosomal subunit proteins by LiCl has been studied earlier using semi-quantitative 2D electrophoresis method [9]. At 0.5 M LiCl, 6 proteins were found to dominate in the core, two of these, rpL8 and rpL25, were also identified as 0.5 M LiCl core proteins in the present study. Overall, the present study identified more proteins in the core at this concentration. The semi-quantitative approach showed that only one protein, rpL25, was found to remain bound to the core at 1 M LiCl [9], whereas in experiments presented here at least three proteins (rpL1, rpL15, and rpL25) were found in the 1 M core by more than 50%. Quantitative MS approach also showed that the amount of some proteins in the core at 0.5 M LiCl approaches 100% while in the 2D electrophoresis study it is estimated to be approximately 50%.

It is interesting to compare the splitting of eukaryotic r-proteins with bacterial ones. As compared to eukaryotes, bacterial r-proteins dissociate at much higher salt concentrations [7], [15]. Although the structure of ribosome is similar in both kingdoms and most of the bacterial r-proteins have eukaryotic homologues, the nature of interactions between rRNA and proteins is considerably different. In general, binding of bacterial r-proteins to rRNA is resistant to higher salt concentration, whereas eukaryotic r-proteins dissociate at significantly lower salt concentrations. This suggests that the impact of ionic interactions between r-proteins and rRNA is more important in the eukaryotic ribosome as compared to the bacterial counterpart.

Small group of bacterial homologues of yeast r-proteins acts similarly in sense of dissociation from ribosomal core following the treatment with LiCl. For example, yeast r-proteins rpS11, rpS13, rpL3 and rpL25 belong to the strongly binding protein group likewise their bacterial homologues S17, S15, L3 and L23, respectively (Table 1) [7]. In addition, some yeast split proteins, e.g. rpL7, rpL9 and rpL11, have bacterial homologues that also belong to the split protein fraction in bacteria.

Few r-proteins however act differently in bacterial and yeast ribosomes. For example, rpL1 is one of the most strongly binding proteins in the yeast ribosome, whereas in bacteria it is one of the most weakly binding proteins. Other differently acting r-proteins are yeast 0.5 M LiCl core proteins rpL10, rpL28 and rpP1/rpP2, whose bacterial homologues, L16, L15 and L12, respectively, belong to the split protein fraction (Table 1) [7]. In addition, some of the most easily dissociating yeast r-proteins (e. g. rpS22, rpL26 and rpL35) have bacterial homologues (S8, L24 and L29, respectively) belonging to the 2 M LiCl core fraction (Table 1) [7]. In conclusion, LiCl splitting results demonstrate that in spite of the sequence homology of the bacterial and yeast r-proteins they often use different binding modes for rRNA.

The splitting-off sequence of the bacterial large subunit r-proteins by increasing LiCl concentrations roughly reflects the assembly process in reverse order [8]. In yeast, it has been shown that few r-proteins (rpS10, rpS26, rpL10, rpL24, rpL29, rpL40, rpL42, rpP0, rpP1 and rpP2) join preribosomes in the cytoplasm during the final stages of the subunits maturation [16]–[20]. These late associating r-proteins dissociate at different LiCl concentrations. For example, rpL10, rpP1 and rpP2 belong to the 0.5 M core fraction, and rpS2, rpL29, rpL40 are the most weakly binding proteins. However, majority of the core proteins at 1 M LiCl are required for the early stages of yeast ribosome biogenesis. For example, rpS14 is the component of the small-subunit processome [21]. Depletion of rpS1 or rpS14 leads to the accumulation of 35S and 23S pre-rRNAs indicating their involvement in early rRNA processing [17]. Down-regulation of expression of rpL3 or rpL25 resulted in inefficient production of pre-rRNAs with a matured end of 5.8S pre-rRNA or delay in the endonuclease cleavage separating 5.8S rRNA and 25S rRNA precursors, respectively [22]. The salt-induced splitting of yeast r-proteins reflects only partially the hierarchical assembly of ribosome subunits unlike it has been observed in bacterial system. However, the assembly essential r-proteins rpS1, rpS14, rpL3, and rpL25 are strongly associated with rRNA according to LiCl splitting.

Of the eukaryote-specific r-proteins only protein rpL15 is significantly associated with the core particles at 1 M LiCl. In addition, 7 eukaryote specific r-proteins (rpL8, rpL13, rpL15, rpL18, rpL19, rpL36, and rpL37) belong to the strongly associated proteins (group A). All other eukaryote-specific r-proteins are removed from the ribosome core already at 0.5 M LiCl. Thus, r-proteins unique to eukaryotes belong mainly to the group of weakly associated proteins.

**Figure 3:**
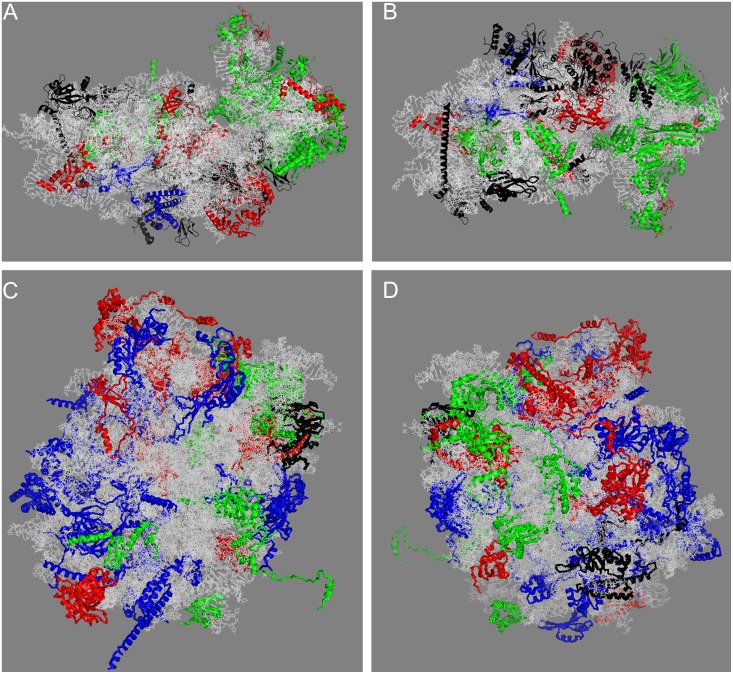
Modeling of split and core proteins in the 3D structure of yeast ribosome subunits. The structures were generated by PyMol using co-ordinates from [1] (**A**) 40S subunit, interface view; (**B**) 40S subunit, solvent side view; (**C**) 60S subunit, interface view; (**D**) 60S subunit, solvent side view. rRNA is shown in gray. Proteins are grouped according to their amount in the core fraction at 0.5 M LiCl. Group A (blue) – 75–100%, B (red) – 48–75%, C (green) – 22–48% and D (black) – 0–22% in the core.

R-proteins specific to eukaryotes are located in the peripheral parts of the ribosome [1], [23], [24]. Easily dissociating bacterial r-proteins are mainly located on the surface of the ribosome or form extended structures such as L7/12 stalk or L1 mushroom [24]. However, labeling of yeast r-proteins according to their LiCl splitting properties (Figure 3) shows that 0.5 M LiCl core proteins do not form any specific region of the ribosome, nor do 0.5 M LiCl split proteins [1]. Moreover, bacterial split proteins were shown to be among the most easily exchangeable proteins both *in vitro* and *in vivo* [25]. Most of the yeast r-proteins are associated with the ribosomal core in a labile way suggesting that the number of exchangeable proteins is bigger for eukaryotic ribosome. It remains to be seen whether the eukaryotic r-proteins are more readily exchangeable as compared to their bacterial counterparts as it could be expected from salt dissociation experiments.

## Supporting Information

Figure S1 **Ribosome subunit profiles after LiCl treatment.** “Light” yeast 80S ribosomes were incubated with indicated concentrations of LiCl and ribosomal particles were analysed by sucrose gradient centrifugation. Sedimentation is from left to right. Lines indicate the location of 80S ribosomes and free 40S and 60S subunits.
(TIF)

Table S1 **The association of yeast r-proteins with rRNA at different LiCl concentrations as determined by quantitative mass spectrometry.** The number of peptides identified for quantitation, average percentage values in core fraction with standard deviations for each r-protein are indicated.
(XLSX)
