# Supplementary figures and images for: Stepwise Splitting of Ribosomal Proteins from Yeast Ribosomes by LiCl
Source: PLoS One. 2014 Jul 3;9(7):e101561. doi: 10.1371/journal.pone.0101561 (PMC4081664; doi:10.1371/journal.pone.0101561)

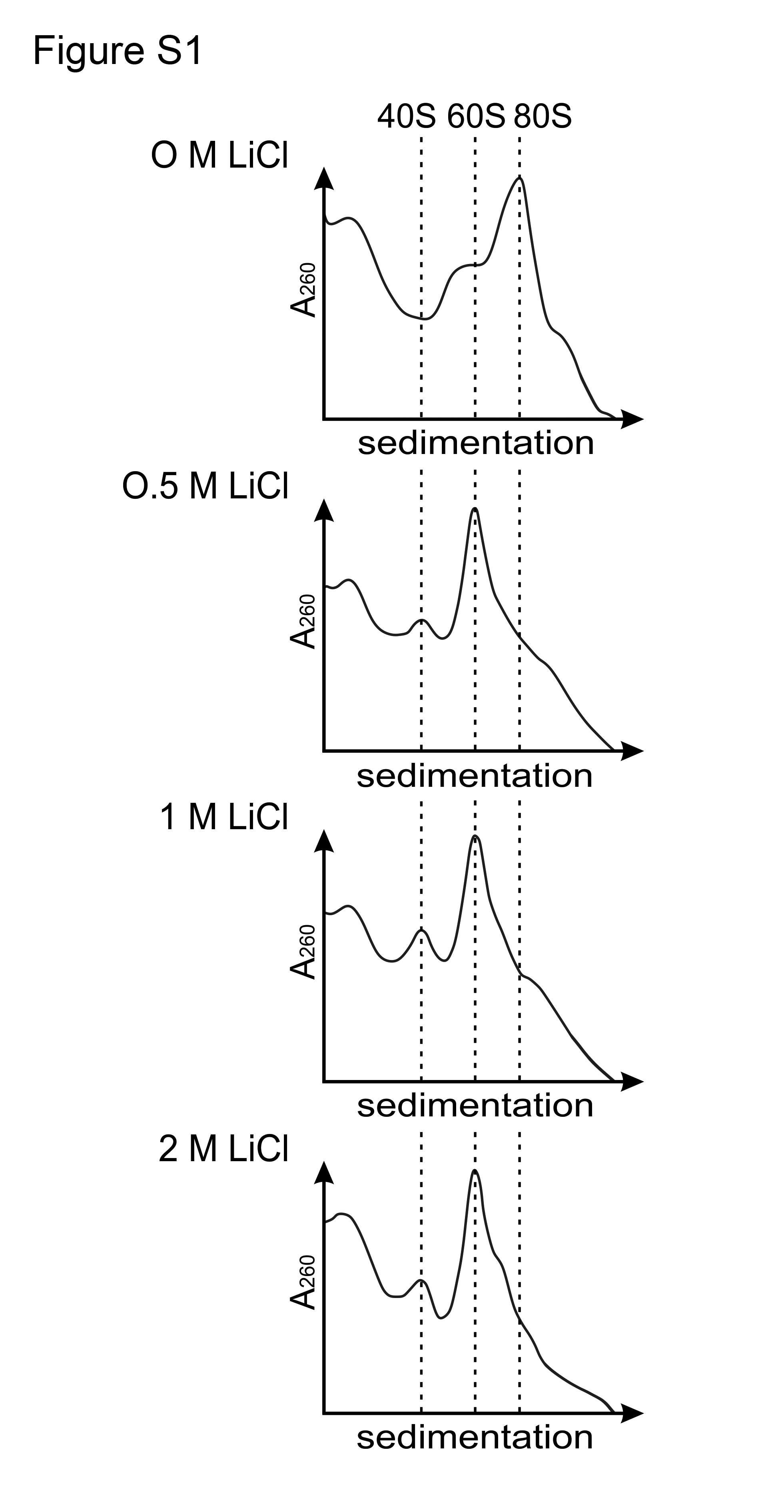

Supplement: Figure S1 — Ribosome subunit profiles after LiCl treatment. “Light” yeast 80S ribosomes were incubated with indicated concentrations of LiCl and ribosomal particles were analysed by sucrose gradient centrifugation. Sedimentation is from left to right. Lines indicate the location of 80S ribosomes and free 40S and 60S subunits. (TIF) [file pone.0101561.s001.tif]
